# The Impact of Parental Screen Time on Children’s Mental Health. Systematic review and meta-analysis

**DOI:** 10.1192/j.eurpsy.2025.452

**Published:** 2025-08-26

**Authors:** A. Leonova, K. Markin, K. Vasilchenko, E. Nemtseva

**Affiliations:** 1 Pain Treatment Clinic, Tyumen; 2 S. M. Kirov Military Medical Academy, Saint Petersburg, Russian Federation; 3 The Human Artificial Control Keren (HacK) lab, Azrieli Faculty of Medicine, Bar-Ilan University, Safed, Israel; 4 Orenburg State Medical University, Orenburg, Russian Federation

## Abstract

**Introduction:**

In the past decade, techno-referentiality has increasingly influenced daily life, illustrating how technology can disrupt human relationships. A notable example of this phenomenon is “phubbing”—a combination of “phone” and “snubbing”—which refers to the act of ignoring someone in favor of engaging with a mobile phone, leading to diminished eye contact and interest during face-to-face interactions. Recent research has highlighted “parental phubbing,” suggesting it may adversely affect parent-child relationships and contribute to developmental and mental health issues in children and adolescents.

**Objectives:**

This study seeks to investigate the potential effects of parental phubbing on the mental health and development of children.

**Methods:**

We conducted a systematic review and meta-analysis using Nested Knowledge software, adhering to PRISMA guidelines. Our search encompassed five databases: PubMed, Scopus, PsycINFO, Web of Science, and EBSCO. The inclusion criteria for studies were:

- Cross-sectional or longitudinal design.
- Quantitative data on parental phubbing and its potential impact on children’s mental health symptoms and developmental disorders.
- Studies involving children aged 0 to 18-21 years.
- Publications in peer-reviewed English-language journals.

The methodological quality and risk of bias in the included studies were assessed using the JBI Critical Appraisal Checklist. Publication bias was evaluated through funnel plot analysis and Egger’s regression intercept. Meta-analyses were performed using Jamovi with the MAJOR module, applying the Fisher r-to-z transformation for correlation coefficients. A random-effects model was used, and heterogeneity was estimated with the restricted maximum-likelihood estimator. Sensitivity analyses were conducted to ensure the robustness of the findings.

**Results:**

Our search identified 26 studies involving 22833 children and 2125 parents. We developed a unified theoretical model of the direct and mediating effects of parental phubbing on child mental health. The meta-analysis revealed that parental phubbing was significantly associated with:

- Increased affective symptoms in children (k=10; r=0.319; 95% CI [0.269, 0.370]).
- Higher levels of aggression or deviant behavior (k=3; r=0.260; 95% CI [0.134, 0.386]).
- Greater internalizing problems (k=4; r=0.242; 95% CI [0.166, 0.319]).
- More externalizing problems (k=4; r=0.158; 95% CI [0.081, 0.234]).

Additionally, parental phubbing was negatively correlated with children’s self-esteem (r=-0.233; 95% CI [-0.315, -0.150]).

**Conclusions:**

This meta-analysis underscores the significant harm that parental phubbing can inflict on children’s emotional and social well-being. To safeguard and enhance children’s development, it is crucial to implement strategies that promote more mindful technology use and foster stronger, more engaged parent-child relationships.

**Disclosure of Interest:**

None Declared

